# Regulation of Tumor Necrosis Factor-related Apoptosis-inducing Ligand Expression in Primary Acute Leukemic Cells by Chemotherapeutics

**DOI:** 10.4274/Tjh.2013.0027

**Published:** 2013-09-05

**Authors:** Shengmei Chen, Yanfang Liu, Hui Sun, Ling Sun, Jie Ma, Dingming Wan, Zhongxing Jiang, Qiutang Zhang, Tao Li

**Affiliations:** 1 Key Observation Laboratory in Henan Medical University, Department of Hematology First Affiliated Hospital, Zhengzhou University, Zhengzhou, Henan Province, P.R. China

**Keywords:** TRAIL, Acute leukemia, chemotherapeutics

## Abstract

**Objective:** The expression of the tumor necrosis factor-related apoptosis-inducing ligand (TRAIL) protein and its regulation by chemotherapeutics were analyzed in primary acute leukemic cells.

**Materials and Methods:** Peripheral blood was collected from 16 patients with acute leukemia on days 0, 1, 3, and 5 of chemotherapy. The mononuclear cells were separated from the peripheral blood, and TRAIL expression was assessed by flow cytometry. The bone marrow mononuclear cells of patients with acute leukemia were separated before chemotherapy and cultured in vitro with VP-16 and/or interferon (IFN). The TRAIL expression level was detected after the cell culture.

Re**sults:** TRAIL expression in the mononuclear cells of peripheral blood was significantly upregulated on day 1 (p<0.05) and then significantly decreased on day 5 after chemotherapy (p<0.05). Results from the in vitro culture revealed that VP-16 upregulated TRAIL expression in the bone marrow mononuclear cells of patients with acute leukemia, but the binding of VP-16 to IFN did not enhance TRAIL expression as compared with VP-16 alone (p>0.05).

**onclusion:** OA single chemotherapy mechanism for leukemia may suffice to induce TRAIL expression and promote the apoptosis of leukemic cells.

**Conflict of interest:**None declared.

## Abstract (tr)

**Amaç:** Primer akut lösemi hücrelerinde, tümör nekrozis faktör alakalı apoptozis indükleyici ligand (TRAIL) protein ekspresyonu ve bunun kemoterapötikler yoluyla kontrolü analiz edildi.

**Gereç ve Yöntemler:** 16 akut lösemi hastasından, kemoterapinin 0, 1, 3 ve 5. günlerinde periferik kan örnekleri toplandı. Mononükleer hücreler periferik kandan ayrılarak akım sitometrik olarak TRAIL ekspresyonu değerlendirildi. Akut lösemili hastaların kemik iliği mononükleer hücreleri kemoterapi öncesi ayrılarak, VP-16 ve/veya interferon (IFN) ile kültive edildi. TRAIL ekspresyonu, hücre kültüründen sonra çalışıldı.

**Bulgular: ** Periferik kandan elde edilen mononükleer hücrelerdeki TRAIL ekspresyonu 1. günde (p<0.05) anlamlı olarak yüksekti ve kemoterapi sonrası 5. gündeyse belirgin olarak düşük saptandı (p<0.05). In vitro kültür sonuçları; VP-16’nın akut lösemili hastaların kemik iliği mononükleer hücrelerindeki TRAIL ekspresyonunu arttırdığını ve fakat tek başına VP-16 ile karşılaştırıldığında IFN ile kombine VP-16’nın TRAIL ekspresyonunu arttırmadığını göstermiştir (p>0.05).

**Sonuç:** Lösemide tekli kemoterapi mekanizmasının TRAIL’i indüklemek ve lösemik hücreleri apoptozise yönlendirmek için yeterli olduğu görülmüştür.

## INTRODUCTION

The process of programmed cell death or apoptosis has a key effect on the occurrence, development, and stability of an organism. This process also has an important role in the inhibition of tumor cell growth and immune surveillance. Aside from the tumor necrosis factor (TNF) and the Fas ligand (FasL), the TNF-related apoptosis-inducing ligand (TRAIL), also known as the apoptosis 2 ligand or Apo2L [1], is a member of the TNF superfamily that induces apoptosis by binding to specific death receptors and activating multiple apoptotic signaling pathways [2]. TRAIL induces apoptosis in tumor cells but has low sensitivity to normal cells [3]. Various key cells of the immune system for tumor surveillance partially depend on TRAIL expression to perform cytotoxic effects and inhibit the occurrence and migration of tumors; these cells include the CD4+ T cells, natural killer (NK) cells, macrophages, and dendritic cells [4]. TRAIL can induce the apoptosis of malignant leukemia cells without harming normal cells [5]. TRAIL expression can also induce the differentiation of surviving leukemic cells and normal myeloid precursors into mature monocytoids and regulate normal hematopoiesis [6]. Liu et al. [7] detected the expression of TRAIL and its receptors in acute myeloid leukemic cells and discovered the high expression of TRAIL, DR4, and DR5 in patients. However, the effects of chemotherapeutics on TRAIL expression in primary acute leukemic cells are rarely reported. Our study used flow cytometry to detect TRAIL expression after incubating acute leukemic cells with chemotherapeutics in vivo and in vitro. We then investigated the significance of TRAIL expression in chemotherapy for acute leukemia. The effects of TRAIL-related drugs were likewise studied. Our study provides a scientific basis for the clinical application of TRAIL. 

## MATERIALS AND METHODS

**Subjects and Specimen Processing **

Blood samples were obtained from 16 patients diagnosed with acute leukemia based on their cytomorphology, histochemistry, and cellular immunology. Peripheral blood was collected from the patients on days 0, 1, 3, and 5 of chemotherapy. Heparin was added to the samples for anticoagulation. Mononuclear cells were separated using a lymphocyte separation medium (Ficoll; density, 1.007) by a standard method, and the concentration of the cells was adjusted to 1x107 cells/L. Bone marrow (10 mL per patient) was collected from 12 patients before chemotherapy. Mononuclear cells were then separated and cultured in a medium containing VP-16 and interferon (IFN). The cells were incubated for 0, 24, 48, and 72 h before they were collected. The final cell concentration was adjusted to 1x107 cells/L with phosphate-buffered saline (PBS; BD, USA). This study was conducted in accordance with the Declaration of Helsinki and with approval from the Ethics Committee of the First Affiliated Hospital, Zhengzhou University. Written informed consent was obtained from all participants. 

**Cell Culture**

The acute leukemic mononuclear cells cultured with VP-16 and IFN in medium (cell concentration, 1x10^7^ cells/L) were divided into 4 groups: the control group, VP-16 group (final VP-16 concentration, 50 µg/mL), IFN group (final IFN concentration, 2000 U/mL), and VP-16+IFN group (same final concentrations as in the VP-16 and IFN groups). The cells were incubated under saturated humidity at 37 °C in an incubator with 5% CO2. 

**Detection of TRAIL Expression by Flow Cytometry**

The initial and cultured cells were divided into 2 tubes each. The first tube had CD45 monoclonal antibodies alone added, whereas the second one contained both CD45 and TRAIL monoclonal antibodies. The TRAIL antibodies were diluted 20-fold in PBS prior to use. Each tube then had 100 µL of cell suspension added. The tubes were incubated in the dark at room temperature for 30 min and washed with PBS 3 times. Afterwards, TRAIL expression was detected by flow cytometry (FACstar; BD, USA). 

The detection results were presented as mean fluorescence intensity (MFI). The MFI of the total mononuclear cells and leukemic cells were recorded as T-MFI and L-MFI, respectively. 

**Statistical Analysis**

The statistical significance of the data was determined using SPSS 10.0. Data were presented as the mean ± standard deviation. The comparison between groups was performed using the paired Student’s t-test. Differences were considered statistically significant at p<0.05. 

## RESULTS

**TRAIL Expression during Chemotherapy**

The T-MFI of TRAIL in patients with initial acute leukemia was significantly increased after chemotherapy, as compared with that before chemotherapy (p<0.01), and then it gradually decreased. The TRAIL expression on day 5 after chemotherapy was significantly decreased as compared with that on day 1 (p<0.05). The L-MFI of TRAIL in patients was distinctly higher on day 1 after chemotherapy than that before the chemotherapy (p<0.01). No significant differences in the TRAIL expression were found among the other days after chemotherapy (p>0.05; Table 1). 

Effect of Chemotherapeutics on the TRAIL Expression in Acute Leukemic Bone Marrow Cells in Vitro 

The T-MFI of TRAIL was enhanced at 24 and 48 h after incubation with both VP-16 and IFN (p<0.05), as compared with that before incubation (p<0.05). L-MFI significantly increased at 24 h after incubation (p<0.01; Table 2). 

**Influence of Different Drugs on TRAIL Expression**

Treatment with VP-16 and the combination of VP-16 with IFN upregulated TRAIL expression in the bone marrow mononuclear cells of patients with acute leukemia at 24 h after the treatment, as compared with that in the control group (p<0.05). IFN induced TRAIL expression in leukemic cells, but its effect was not statistically significant (p>0.05). The TRAIL expression level induced by IFN was distinctly different from that induced by the combination of IFN and VP-16. No pronounced changes in the TRAIL expression level were observed between the incubation with VP-16 alone and the drug combination (p>0.05; Table 3). 

## DISCUSSION

Plasilova et al. [5] reported that TRAIL can induce the apoptosis of leukemic cells such as K562, HL-60, and ML-1 in a dose-dependent manner. TRAIL reduces the formation of myeloid colony forming unit-granulocyte/macrophage (CFU-GM) colonies in patients with acute non-lymphocytic leukemia (ANLL), chronic myeloid leukemia (CML), and myelodysplastic syndromes, as well as inhibits cell growth. However, the growth and proliferation of selected cells are not affected by TRAIL expression, which includes the cells in ANLL patients with completely remitted blood, cells in lymphoma patients with unaffected bone marrow, normal bone marrow precursors, and normal umbilical cord blood precursors. Lee et al. [8] treated a mixture of cord blood mononuclear cells and Jurkat cells with TRAIL. They found that Jurkat cells are specifically eliminated without decreasing the number of normal CFU-GM colonies. A previous study showed that most Ph(+) leukemic cells are resistant to the apoptosis induced by FasL but highly sensitive to TRAIL [9]. The death receptors DR4 and DR5 are expressed on the surface of these TRAIL-sensitive cells. However, these receptors are expressed in low levels or are absent in TRAIL-resistant cell lines. Therefore, the sensitivity of leukemic cells to TRAIL depends on the expression of death receptors on their surfaces. The induction of apoptosis has an important role in monitoring tumor immunity and chemotherapy by increasing the expression of TRAIL and its specific death receptors as well as their interaction. In this study, we utilized flow cytometry to investigate the effect of chemotherapy on TRAIL expression in the leukemic cells of patients with initial acute leukemia. The results showed that the T-MFIs of TRAIL on days 0, 1, 3, and 5 after chemotherapy were 3.20±0.76, 3.86±0.88, 3.91±1.22, and 3.29±0.88, respectively. T-MFI significantly increased on day 1 after chemotherapy as compared with that before chemotherapy (p<0.01). However, no obvious differences in TRAIL expression were found between day 0 and days 3 or 5. The TRAIL expression level on day 5 after chemotherapy was significantly decreased as compared with that on day 1 after chemotherapy (p<0.05). No significant differences in TRAIL expression were observed among the other days (p>0.05). The L-MFIs of TRAIL in acute leukemia patients were 3.59±1.23, 4.05±1.53, 4.34±1.77, and 3.91±1.31 on days 0, 1, 3, and 5, respectively. L-MFI was significantly higher on day 1 after chemotherapy than before the chemotherapy (p<0.01). No significant differences in TRAIL expression were found among the other days (p>0.05). Chemotherapeutics function by inducing the apoptosis of leukemic cells. TRAIL is a type II transmembrane protein that induces apoptosis, which maintains cell homeostasis in normal cells and has cytotoxic effects on tumor cells. This study reveals that chemotherapeutics could promote TRAIL expression in leukemic cells. TRAIL expression eventually decreased with prolonged chemotherapy. Thus, chemotherapy may have improved TRAIL expression, which induced the apoptosis of the acute leukemic cells. 

Previous studies showed that IFN-α can upregulate TRAIL expression on the surfaces of active T cells [9]. Allogeneic stem-cell transplantation (Allo-SCT), as a curative treatment option for acute leukemia and chronic granulocytic leukemia, mainly eliminates leukemic cells via the immune-mediated graft-versus-leukemia (GVL) effect. Shlomchik and Pear [10] reported that the GVL effect is not reduced in Fas-deficient mice, which implies that the TRAIL/death receptor interaction might influence the elimination of immune-mediated CML malignant cloning. Furthermore, FasL/Fas interaction is not the main signaling pathway for inducing apoptosis. Wen et al. [11] revealed that anti-leukemia drugs such as Ara-C and daunorubicin promote DR5 expression in leukemic cell lines and induce a cytotoxic effect. This study investigated TRAIL expression in mononuclear cells of acute leukemia patients in complte remission. We discovered that TRAIL expression was significantly increased on day 1 after chemotherapy as compared with that before the chemotherapy (p<0.05). However, no obvious differences in TRAIL expression were found between day 0 and days 3 or 5. The TRAIL expression level on day 5 after chemotherapy was significantly decreased as compared with that on day 1 after chemotherapy (p<0.05). No significant difference in TRAIL expression was found among the other days studied (p>0.05). However, the TRAIL expression in leukemic cells and CD8+ T cells was not significantly changed (p>0.05). These results demonstrated that TRAIL expression did not increase in leukemic cells and CD8+ T cells of patients in complete remission. The elimination of residual leukemic cells did not rely on TRAIL expression during the consolidation phase of treatment. The chemotherapy enhanced TRAIL expression in all mononuclear cells. We hypothesize that the increased TRAIL expression was not observed in the leukemic and CD8+ T cells, but it was the case in other mononuclear cells. The chemotherapy may have partially depended on the TRAIL expression of immune cells such as NK cells and macrophages to kill the residual leukemic cells. Subsequently, TRAIL binds to the death receptors to activate the apoptosis signaling pathway. 

The combination of Ara-C and IFN-α remarkably increases the remission rate of CML patients and prolongs patient survival [12]. Kanako compared the anti-leukemia effect between TRAIL and STI-571 and found that malignant myeloid cells, as well as a type of malignant lymphocyte, are the most sensitive to STI-571 among the 8 types of malignant lymph cell lines. Four naturally STI-571-resistant cell lines are sensitive to TRAIL, whereas 5 STI-571-sensitive cell lines are resistant to TRAIL. However, all the tested cell lines are highly sensitive to the combination of TRAIL and STI-571. In our study, the bone marrow mononuclear cells of patients with acute leukemia were treated with VP16 and IFN. The T-MFIs of TRAIL were 3.44±1.12, 5.12±1.20, 6.42±2.73, and 6.20±3.32 at 0, 24, 48, and 72 h of treatment, respectively. The T-MFI of TRAIL was enhanced at 24 and 48 h after incubation with VP-16 and IFN (p<0.05), as compared with that before incubation (p<0.05). By contrast, L-MFI was 3.39±0.94, 5.38 ± 1.01, 5.26±1.98, and 4.04±1.36 at 0, 24, 48, and 72 h of incubation, respectively. L-MFI was significantly increased at 24 h after incubation (p<0.01). No significant differences in L-MFI were found among the other treatment times (p>0.05). 

VP16 is a topoisomerase II inhibitor commonly used in chemotherapy protocols for acute leukemia to induce the apoptosis of leukemic cells. IFN is an antitumor cytokine involved in immune regulation by inhibiting cell hyperplasia. The combination of these 2 drugs can enhance TRAIL expression in leukemic cells, which is consistent with the observation that chemotherapy promotes TRAIL production in leukemic cells. These drugs can kill leukemic cells and induce apoptosis by promoting TRAIL expression. 

To further study the effects of these drugs, the cells were divided into 4 groups according to drug treatment. The results showed that the T-MFIs of TRAIL in the control, VP-16, IFN, and VP-16+IFN groups were 5.05±0.89, 6.32±1.68, 5.35±1.28, and 6.74±2.15, respectively, with the corresponding L-MFIs of 5.02±1.13, 6.06±1.73, 5.41±1.40, and 6.36±1.55. VP-16 as well as the combination of VP-16 with IFN upregulated TRAIL expression in the bone marrow mononuclear cells of patients with acute leukemia compared with that in the control group (p<0.05). However, IFN alone did not cause any significant change (p>0.05). The TRAIL expression level induced by IFN was distinctly different from that induced by the combination of IFN and VP-16 (p<0.05). No obvious changes in the TRAIL expression levels were observed between the incubation with VP-16 alone and the drug combination (p>0.05). This result strongly suggested that TRAIL expression is primarily regulated by V16 but not by IFN. This observation is not consistent with the published literature, which indicates that IFN-α can upregulate TRAIL expression on the T cell surface. This difference may be related to the different culture systems used in our experiments. Furthermore, the mononuclear cells cultured in our experiment were primary leukemic cells, with only a small proportion of immune cells. Thus, IFN alone had a weak effect on inducing TRAIL expression and promoting apoptosis. 

It is reported that IFN and its therapeutic applications have serious side effects, such as the lethal graft-versus-host disease effect of Allo-SCT and resistance to STI-571 in CML treatment. These side effects limit the effectiveness and persistence of these therapeutic option [13]. Only 2 out of 19 acute myeloid leukemia cases demonstrated leukemia cell apoptosis (>10%) after TRAIL treatment on mononuclear cells alone. However, the effect increases when TRAIL treatment is combined with fludarabine, cytarabine, or daunorubicin. Nearly half of the mononuclear cells underwent apoptosis under this treatment, and the proapoptotic effect was as strong as that of caspase-8. Conticello et al. [14] confirmed that bortezomib with TRAIL induces the expression of TRAIL and its receptor to improve the sensitivity to the treatment. With the increased binding of TRAIL and the death receptors, more leukemia cells are subjected to apoptosis. TRAIL is characterized by a specific anti-leukemia effect, which makes it a promising therapy for leukemia. The role of TRAIL receptors in the TRAIL-induced apoptotic pathway still needs to be further explored. Given the limited number of reports to date, more cases are necessary to confirm the conclusions drawn from this study. 

Various forms of recombinant TRAIL proteins have been successfully developed. Human Jurkat cells are more sensitive to the recombinant leucine zipper–TRAIL [15], which induces the apoptosis of human breast cancer MDA-231 cells without harming normal breast epithelium cells and other normal tissues. The continuous and systematic injection of excess TRAIL to non-human primates does not change the clinical and histopathological index. This finding demonstrates the safe application of TRAIL in primates, which indicates progress in the eventual application of TRAIL in humans. Ichikawa et al. [16] produced a specific DR5 monoclonal antibody, TRA-8, which is sensitive to primary and metastatic liver cells and specifically binds to DR5 without inducing the apoptosis of normal liver cells. In vitro and in vivo animal experiments show the cytotoxic effect of other proteins, such as the recombinant isoleucine zipper–TRAIL (hFlex-TRAIL) [17] and the plasmid pRevTRE-TRAIL [18]. We think that the clinical application of recombinant human TRAIL to human malignant tumors, including leukemia, can enhance the sensitivity of chemotherapeutics and increase the GVL effect of Allo-SCT, as well as reduce the required drug dose and the side effects, thereby effectively improving the leukemia treatment and patient prognosis. 

## CONCLUSION

Meeting the needs of hematologists for more education and knowledge on the topic of fertility preservation and improving their knowledge, attitudes, and practices in this area is of great importance. The Turkish Society of Hematology may play a significant and key role in such efforts. 

## CONFLICT OF INTEREST STATEMENT

The authors of this paper have no conflicts of interest, including specific financial interests, relationships, and/ or affiliations relevant to the subject matter or materials included. 

## Figures and Tables

**Table 1:**
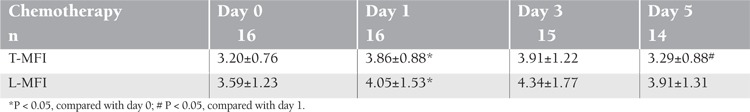
The TRAIL expression in primary acute leukemic cells during chemotherapy

**Table 2:**
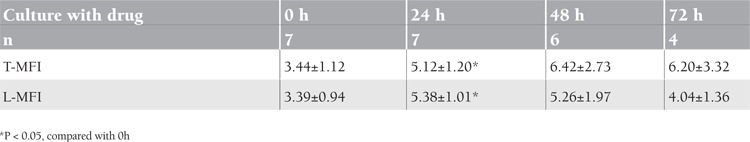
The TRAIL expression in acute leukemic cells treated with VP-16 and INF for different time

**Table 3:**
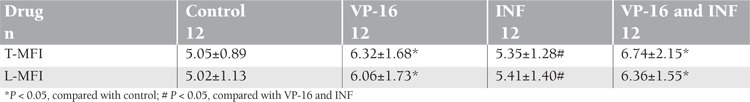
The TRAIL expression in acute leukemic cells treated with different drugs.
